# Supplementary material for: The genome sequence of the Knapweed Pearl, Paratalanta hyalinalis (Hübner, 1796) (Lepidoptera: Crambidae)
Source: Wellcome Open Res. 2025 Oct 6;10:548. [Version 1] doi: 10.12688/wellcomeopenres.24962.1 (PMC12627939; doi:10.12688/wellcomeopenres.24962.1)
Supplement: Peer review history

# Reviewer report: Reviewer response for version 1

**DOI:** 10.21956/wellcomeopenres.27497.r137314

**Published:** 2025-11-18

**Authors:** João Pimenta

**Affiliations:** 1 CIBIO-InBIO, Research Centre in Biodiversity and Genetic Resources, Porto, Portugal

This genome note is overall well-structured, follows the standard Darwin Tree of Life reporting format, and presents a high-quality genome assembly for *Paratalanta hyalinalis*. The content is technically detailed and methodologically clear. Background information on the species is extensive and informative. 

 The Methods section is comprehensive and follows DToL protocols. The assembly pipeline description is complete and up to current standards.

 Comments:

 The authors mention that sample metadata was collected following DTol standards. However, there is no indication of where the metadata can be accessed. I'm aware that the metadata is deposited in COPO and accessible through ENA, following Lawniczak et al. (2022), but a sentence should be added to clarify where the data has been deposited and how It can be accessed.

 Typos:

 ha plotype - Background section - "…identical to the most common ha plotype on BOLD…”

 There are some italics missing from species names throughout the text

 "In the Palaearctic the species ranges from Great Britain (excluding Ireland)..." - comment: Great Britain already excludes Ireland, maybe change to: "British Isles (excluding Ireland)"

Are sufficient details of methods and materials provided to allow replication by others?

Yes

Is the rationale for creating the dataset(s) clearly described?

Yes

Are the datasets clearly presented in a useable and accessible format?

Yes

Are the protocols appropriate and is the work technically sound?

Yes

Reviewer Expertise:

Evolutionary genomics.

I confirm that I have read this submission and believe that I have an appropriate level of expertise to confirm that it is of an acceptable scientific standard.

# Reviewer report: Reviewer response for version 1

**DOI:** 10.21956/wellcomeopenres.27497.r135540

**Published:** 2025-11-03

**Authors:** Yvonne Jing Mei Liew

**Affiliations:** 1 Universiti Malaya, Kuala Lumpur, Kuala Lumpur, Malaysia

1. Correct typo haplotype - and is identical to the most common ha plotype on BOLD (31/07/2025).

 2. Figure 1 - a scale should be provided to indicate the size of the specimen

 3. Table 3 should include the information for mitochondrial genome sequence

 4. Can the author explain the reason why mitochondrial genome is not assembled from Illumina sequence data for Haplotype 2?

Are sufficient details of methods and materials provided to allow replication by others?

Yes

Is the rationale for creating the dataset(s) clearly described?

Yes

Are the datasets clearly presented in a useable and accessible format?

Yes

Are the protocols appropriate and is the work technically sound?

Yes

Reviewer Expertise:

Genomics (mitogenome, NGS)

I confirm that I have read this submission and believe that I have an appropriate level of expertise to confirm that it is of an acceptable scientific standard.
